# Acaricidal Properties of Bio-Oil Derived From Slow Pyrolysis of *Crambe abyssinica* Fruit Against the Cattle Tick *Rhipicephalus microplus* (Acari: Ixodidae)

**DOI:** 10.3389/fphys.2021.768522

**Published:** 2021-12-02

**Authors:** Camila Mattos, Juliana Andrade, Bruno Salarini Peixoto, Nayara Luiza Tavares Moraes, Marcia Cristina da Cunha Veloso, Gilberto Alves Romeiro, Evelize das Chagas Folly

**Affiliations:** ^1^Laboratory of Pest and Parasite Studies, Department of Cellular and Molecular Biology, Institute of Biology, Fluminense Federal University, Niterói, Brazil; ^2^Postgraduate Program in Science and Biotechnology, Fluminense Federal University, Niterói, Brazil; ^3^Laboratory of Synthesis, Chromatography, and Environment, Department of Organic Chemistry, Institute of Chemistry, Fluminense Federal University, Niterói, Brazil; ^4^Postgraduate Program in Chemistry, Fluminense Federal University, Niterói, Brazil; ^5^National Institute of Science and Technology, Molecular Entomology, Rio de Janeiro, Brazil

**Keywords:** slow pyrolysis, bio-oil, crambe fruit, acaricidal activity, cattle tick, ecofriendly

## Abstract

Slow pyrolysis is a process for the thermochemical conversion of biomasses into bio-oils that may contain a rich chemical composition with biotechnological potential. Bio-oil produced from crambe fruits was investigated as to their acaricidal effect. Slow pyrolysis of crambe fruits was performed in a batch reactor at 400°C and chemical composition was analyzed by gas chromatography-mass spectrometry (GC-MS). The bio-oil collected was used in bioassays with larvae and engorged females of the cattle tick *Rhipicephalus microplus.* Biological assays were performed using the larval packet test (LPT) and adult immersion test. The GC-MS of crambe fruit bio-oil revealed mainly hydrocarbons such as alkanes and alkenes, phenols, and aldehydes. The bio-oil in the LPT exhibited an LC90 of 14.4%. In addition, crambe bio-oil caused female mortality of 91.1% at a concentration of 15% and a high egg-laying inhibition. After ovary dissection of treated females, a significant reduction in gonadosomatic index was observed, indicating that bio-oil interfered in tick oogenesis. Considering these results, it may be concluded that slow pyrolysis of crambe fruit affords a sustainable and eco-friendly product for the control of cattle tick *R. microplus.*

## Introduction

A major concern in livestock is the infestation in cattle by the tick *Rhipicephalus microplus* (Canestrini and Fanzago, 1887) (Acari: Ixodidae). This tick can be found in different places in the world with a tropical and subtropical climate and is responsible for a loss of 22–30 billion dollars a year in the livestock industry (Lew-Tabor and Valle, 2016; Ali et al., 2019). Blood spoliation by *R. microplus* causes a reduction in weight gain of bovine and, consequently, decreases the production of meat and milk. In addition, this tick is a vector of infectious agents that cause babesiosis and anaplasmosis (Araújo et al., 2015).

The most used strategy for the control of *R. microplus* is using synthetic acaricides. However, these acaricides are mostly toxic to humans, animals, and the environment. In addition, resistance to acaricides has been reported and has become one of the major obstacles in tick control programs. There are populations of ticks with multiple resistance, with cases of resistance to six classes of acaricides also to associations (Fernández-Salas et al., 2012; Reck et al., 2014; Higa et al., 2015; Klafke et al., 2017). Therefore, it becomes necessary to develop new products for the control of *R. microplus* that are safe for the environment, human, and animal health, and have a low cost.

A new area that has been investigated is the use of products pyrolysis from plant biomass, which has a high biotechnological potential. Plant biomass and agroindustrial residues can be thermochemically converted by pyrolysis. Pyrolysis is a process where an organic matter is subjected to high temperatures (300–1,000°C) in the absence of oxygen (Bridgwater, 2012), generating as products: biochar, pyrolytic liquid, and combustible gases. Pyrolytic liquid can be separated by density difference in an aqueous fraction and a bio-oil (organic fraction) (Czernik and Bridgwater, 2004; Kraiem et al., 2016). The composition of bio-oils varies with the biomass used, being a complex mixture of organic compounds with different chemical functions. Bio-oils obtained from lignocellulosic biomass contain phenolic derivatives, hydroxyaldehydes, hydroxyketones, sugars, and carboxylic acids, mainly acetic acid and formic acid. This chemical composition is due to the depolymerization and fragmentation reactions of the three main constituents of plant biomass: cellulose, hemicellulose, and lignin (Mohan et al., 2006).

Pyrolytic liquids have been the subject of studies for several purposes, mainly as an alternative to fuel production. In recent years, some research groups have revealed the biopesticidal effect of bio-oils against organisms such as insects, bacteria, and fungi (Mattos et al., 2019). The pyrolysis process has great socioenvironmental potential, for generating renewable products and having a reduced emission of greenhouse gases (Silva et al., 2014).

Crambe (*Crambe abyssinica* Hochst) is an annual herbaceous plant of the Brassicaceae family, native to eastern Africa and the Mediterranean region, which has a low production cost and high tolerance to grow in different climatic conditions (Falasca et al., 2010). Crambe fruit is a small sphere-shaped siliqua about 2 mm in diameter, consisting of a thin pericarp and a single seed that is covered by a thin brown husk. The pericarp, which remains attached to the seed, possesses around 30% of the total mass of fruit, has a high content of lignin (40%) and cellulose (41%). The fruit contains about 21% protein, 16–18% fiber, and 30–44% of a nonedible oil that has a high content of erucic acid (C_22:1_), a raw material to produce industrial lubricants, synthetic rubber, plastic films, nylon, and adhesives (Falasca et al., 2010; Ionov et al., 2013; Hu et al., 2015; Bassegio et al., 2016; Samarappuli et al., 2020).

Considering the need for alternatives to control the cattle tick *R. microplus*, the study of the biotechnological potential of pyrolysis bio-oil can provide the development of new products with acaricidal properties. This work aimed to determine the chemical composition of bio-oil derived from slow pyrolysis of crambe fruit, and the acaricidal activity on larvae and engorged females, and the effect on reproduction of *R. microplus*.

## Materials and Methods

### Biomass and Bio-Oil Production

Crambe fruits were supplied by the MS Foundation (Maracajú, Mato Grosso do Sul, Brazil; 21° 37′49″ S, 55°09′37″ W). The bio-oil from crambe fruit (dry fruit consisting of the pericarp, seed, and husk) was obtained through the slow pyrolysis carried out in a batch reactor of the Laboratory of Synthesis, Chromatography, and Environment (SINCROMA). The reactor consists of: A Heraeus R/O 100 oven; a fixed bed of borosilicate type glass with ground joints, dimensions 1.40 cm × 10 mm; a temperature controller and operating time; a liquid collection system consisting of a condenser, a settling funnel (500 ml) and gas scrubber flasks.

Fifty grams of crambe fruit were subjected to slow pyrolysis at 400°C. The biomass was placed in the central region of a cylindrical glass tube which was introduced into the reactor and connected to the condensation system. Nitrogen gas was continuously applied, 500 ml/min, before and throughout the process. The sample was subjected to a heating rate of 10°C/min and the temperature was then held at 400°C for 2 h. The pyrolytic biochar was trapped in the middle of the reactor and collected after cooling. The non-condensable gases passed through a gas scrubber system and were bubbled in water. The condensable gases, when passing through a condenser, generated the pyrolytic liquid. The liquid fractions were separated from the organic phase (bio-oil) by density difference in a separating funnel.

### Gas Chromatography-Mass Spectrometry (GC-MS) Analysis

The characterization of bio-oil was carried out by gas chromatography coupled to mass spectrometry (GC-MS), after fractionation by classical liquid chromatography, using hexane and dichloromethane eluents.

The analyzes by GC-MS were carried out in a Shimadzu apparatus (QP2010), automatic sampler QP, coupled to the Mass Spectrometer, using a fused silica capillary column DB5-MS (20 m × 0.18 mm in internal diameter, 0.18 μm of phenyl polydimethylsiloxane). Helium was employed as a carrier gas at a flow rate of 0.6 ml min^–1^ and the injector (mode split, 1:10) temperature was 280°C. The initial oven temperature was 40°C (5 min hold) and was ramped to 230°C at 5°C min^–1^ and 230°C during 10 min. The mass detector was operated under the following conditions: the temperature at 250°C, electron ionization energy, 70 eV; scan range, 40–600 Da; MS interface temperature were maintained at 200°C.

Compounds were identified by comparing mass spectra with the National Institute of Standards and Technology 147 mass spectral library, considering a similarity equal to or greater than 85%. The semiquantitative analysis was done by normalizing the areas of the identified substances.

### Preparation of Samples for Acaricidal Test

Crambe fruit bio-oil dissolved in an aqueous solution with 5% of tween 80 (v/v) as an emulsifying agent. Solutions were prepared to obtain the concentrations of 25, 20, 15, 10, and 5% (v/v) for larvae test and 25, 15, and 10% (v/v) for female test. The emulsifying solution (5% tween 80) and distilled water were used as the negative controls. Commercial products containing amitraz (Triatox/MSD saúde animal) and deltamethrin (Butox/MSD saúde animal) were used as positive controls at concentrations of 250 and 25 μg/ml, respectively.

### Ticks for Bioassays

*Rhipicephalus microplus* ticks of the Porto Alegre, Brazil strain were maintained on infested Hereford bovines acquired from a tick-free area. All bovines were housed in individual tick-proof pens on slatted floors in the Faculty of Veterinary from the Federal University of Rio Grande do Sul, Porto Alegre (UFRGS) and Institute of Veterinary Research Desidério Finamor (FEPAGRO), Brazil (Reck et al., 2009; Ali et al., 2016). After the cycle on the host is completed, fully engorged females dropped from calves were thoroughly washed with tap water and dried on a filter paper towel. Part of the engorged females was used for the adult immersion test (AIT), while others were kept in a bio-oxygen demand (BOD) incubator at 28°C and 70–80% relative humidity (RH) for approximately 20 days to obtain eggs and larvae, which were later used in biological assays.

All experiments were conducted following the guidelines of the Ethics Committee on Animal Experimentation of UFRGS and FEPAGRO, Brazil (institutional approval number 14403).

### Larval Packet Test (LPT)

The larval packet test (LPT) was performed following the methodology defined by FAO (2004). The filter paper packages (3 × 3 cm) were impregnated with 180 μl solution uniformly distributed with a pipette on both sides. About 100 tick larvae, aged around 14–21 days old, approximately, were added to each filter paper package, and the ends were sealed with a staple. The packets were placed in a BOD incubator at 28°C and 70–80% RH for 24 h. After 24 h, the envelopes were opened and inspected using a stereoscope, to record the number of live and dead larvae. Larvae with walking ability are considered alive. Larvae that are immobile or that move but cannot walk are classified as dead. The test was repeated three times with different batches of larvae and performed in duplicate.

### Adult Immersion Test (AIT)

The AIT was performed as described by Drummond et al. (1973) with minor modifications. Ticks were distributed to groups randomly (15 engorged females per group). The groups of *R. microplus* were immersed for 1 min in 3 ml solution of the respective concentrations (25, 15, and 10%) of bio-oils. After this period, ticks were removed from the solution with the aid of a sieve, distributed in Petri dishes (9 cm diameter, 1.5 cm high), weighed, and kept in a BOD incubator at a temperature of 28°C and 70–80% RH. The mortality of the females was evaluated daily for 15 days. The dead ticks were diagnosed using parameter increasing cuticle darkness, hemorrhagic skin lesions, and stopped Malpighian tube movement observed in a stereomicroscope (Pirali-Kheirabadi et al., 2009). After 15 days, the eggs laid were placed in a glass tube, weighed, and observed separately, using the same condition of incubation for the next 30 days for visual estimation of hatching rate. This experiment was performed three times in duplicate.

The percentage inhibition of oviposition (IO) was calculated according to Singh et al. (2015), as follows:

Reproductive index (RI) = average weight of eggs laid (mg)/average weight of females before treatment (g).
Percentage inhibition of oviposition (IO%) = (RI of the control group – RI of treated group/RI control group) × 100.

### Morphometry of Ovaries

Engorged females of *R. microplus* were incubated with crambe fruit bio-oil solution at a concentration of 25%, following the methodology of AIT, and then kept in a BOD incubator, with 70–80% RH and 28°C. The ticks were subsequently dissected 24, 48, and 72 h after immersion, with the aid of a stereomicroscope, using a 0.01 M phosphate-buffered saline solution. The ovary was removed and weighed. A total of 45 females/treatments were used.

To quantify the interference of pyrolysis bio-oil in the development of the ovary, the gonadosomatic index (GSI) was calculated by dividing the total weight of the ovary by the mean body weight of each group of females, both in the control and treated groups, for each period evaluation (Barbosa et al., 2016).

The percentage inhibition of ovarian development (IOD%) was calculated as follows:


$$\%\text{IOD}=\frac{\left(\mathrm{GSI}\,\mathrm{control}\,\mathrm{group}-\mathrm{GSI}\,\mathrm{treated}\,\mathrm{group}\right)}{\mathrm{GSI}\,\mathrm{control}\,\mathrm{group}}\times 100$$

### Statistical Analysis

Data were expressed as the mean ± SD of the mean. The efficacy was assessed by measuring tick mortality (%) and the lethal concentrations for 50% (LC50) and 90% (LC90) with their 95% confidence limits values were estimated by applying regression equation analysis to the probit data of mortality.

Groups were compared using the one-way ANOVA and the Tukey test. A *p* value less than 0.05 was considered significant. Statistical analysis was performed using GraphPad Prism 6.0 software.

## Results

### Bio-Oil Composition

The yield of coal and pyrolysis liquids were estimated from the mass of each product concerning the mass of raw material, and yield values of the gaseous product were obtained by difference. The yield of crambe fruit pyrolysis products is related to the mass of each product compared to the mass of the raw material used in the process, and the yield values of the gaseous products were obtained by difference. The pyrolysis of the crambe fruit generated a higher bio-oil yield than the aqueous fraction (Table 1). The total mass yield of bio-oil was 34% (w/w). The mains organic functions identified by GC-MS of compounds present in bio-oil were: hydrocarbons, phenol, aldehydes, heterocyclic nitrogenous, ketones, nitrile, amides, and ester. Analysis of hexane fraction showed the presence of substances derived mainly from pyrolysis of triacylglycerides contained in the seed, such as linear alkanes, alkenes, and alkynes, alkadienes, mononuclear alkylbenzenes, aldehydes, carboxylic acids, and esters. All alkanes and alkenes identify in this fraction had similarities above 95%. High molecular mass nitriles (C_16_ – C_19_) were also identified in this fraction. Semiquantitative analysis of hexane fraction performed by normalizing the areas of all identified compounds showed that major compounds were: heneicos-9-ene, from the thermochemical conversion of erucic acid; heneicos-1-ene; hexadec-11-al; heptadec-1-ene; and heneicosane. We were identified this compounds in a small proportion a phytosterol (β-tocopherol), high mass molar hydrocarbons, and steroids as ergostenol, ergostene, stigmastenol, and cholestanone. Some of these substances have already been identified in crambe fruit bio-oil (Silva et al., 2019). In fraction eluted with dichloromethane, the predominance was phenols and methoxy-phenols from the thermal decomposition of the lignocellulosic part of the pericarp and N-heterocycles compounds such as pyridines, pyrroles, and pyrazines. Nitrogen compounds come from the pyrolysis of crambe fruit seed proteins. Phenolic compounds were the majority in this fraction (Table 2).

**TABLE 1:** The yield of crambe fruit pyrolysis products.

	**Yield (%)**			
	**Biochar**	**Bio-oil**	**Aqueous phase**	**Gas**
Crambe	37	34	20	9

**TABLE 2:** Identification and quantification by GC-MS of the main compounds present in the crambe fruit bio-oil.

**Compounds**	**RI**	**Area (%)**
**Dichlromethane**		
2-ethylpyridine	3.26	2.38
2-furylmethylketone	3.33	1.25
ethylpyrazine	3.54	2.60
3-methyl-2-cyclopenten-1-one	4.26	2.34
phenol	4.55	22.40
3-methoxy-pyridine	4.95	2.63
2,3-dimethyl-2-cyclopenten-1-one	5.70	3.05
2-acetylpyrrole	6.37	2.07
4-methylphenol	6.65	32.50
2-methoxyphenol	6.89	1.62
1-methyl-2,5-pyrrolidinedione	7.04	3.54
4-ethylphenol	8.93	5.79
2,6-dimethoxyphenol	13.61	2.93
O-methyloxime-decanal	32.23	4.36
N,N-dimethyldecanamide	32.86	1.21
O-methyloxime-tetradecanol	35.69	2.70
N,N-dimethyl-9-octadecenamida	35.92	2.08
**Hexane**		
tetradecane	15.07	1.12
pentadecane	17.57	2.20
hexadec-1-eno	19.57	1.19
hexadecane	19.94	1.22
heptadec-8-ene	21.69	2.98
heptadec-1-ene	21.84	6.00
heptadecane	22.23	2.10
octadec-3-ene	23.99	1.66
octadecane	24.36	1.31
nonadec-9-ene	25.88	1.54
nonadec-1-ene	26.06	2.73
nonadecane	26.44	2.80
eicos-1-ene	27.88	1.38
eicos-9-ene	28.03	2.07
eicosane	28.39	1.80
heneicos-9-ene	29.79	7.13
heneicos-1-ene	29.99	14.65
heneicos-3-ene	30.16	1.03
heneicosane	30.33	5.93
methyl octadec-9-enoate	30.38	1.52
*E*-hexadec-11-al	37.02	7.70
methyl docos-13-enoate	37.07	1.08
erucic acid	37.17	1.23
octadecanitrile	37.30	2.38

*RI, retention index on DB-5MS column in reference to *n* alkanes.*

### Acaricidal Bioassay With Ticks Larva

The acaricidal effect of bio-oils was tested using larvae of *R. microplus*. In LPT it was observed that the crambe fruit bio-oil, in all concentrations, caused significant mortality of *R. microplus* larvae 24 h after treatment. At a concentration of 15%, crambe bio-oil reached 91.6% of larval mortality (Table 3). The commercial acaricide amitraz was not effective against larvae and deltamethrin caused low mortality of larvae (45%). The calculation of regression analysis of larvae showed an LC50 and LC90 of 7.6 and 14.4%, respectively (Table 4).

**TABLE 3:** Evaluation of the larval packet test of *Rhipicephalus microplus* after treatment with pyrolysis bio-oil of crambe fruit.

**Treatments (%)**	**LM (%) ± SD**
**crambe bio-oil**	
25	99.0 ± 1.1^a,b,c^
20	94.0 ± 3.5^a,b,c^
15	91.6 ± 8.9^a,b,c^
10	84.8 ± 14.0^a,b,c^
5	14.4 ± 7.0^c^
Amitraz	1.5 ± 2.6
Deltamethrin	45.1 ± 13.6^a,b^
Tween 80 5%	0.0 ± 0.0
Water	0.0 ± 0.0

*LM (%): percentage of larval mortality after 24 h.*

*^*a*^ Significant difference in relation to negative controls (water and 5% Tween 80).*

*^*b*^ Significant difference in relation to the positive control Amitraz (250 μg/ml).*

*^*c*^ Significant difference in relation to the positive control Deltamethrin (25 μg/ml) one-way ANOVA *p* = < 0.0001.*

**TABLE 4:** Lethal concentration of 50% (LC50) and 90% (LC90) obtained through larval packet test of *Rhipicephalus microplus* treated with bio-oil, 24 h after treatment.

**Treatments**	**LC50 (%)**	**LC90 (%)**
crambe fruit bio-oil	7.6	14.4

### Acaricide Bioassays With Ticks Female

**FIGURE 1:**
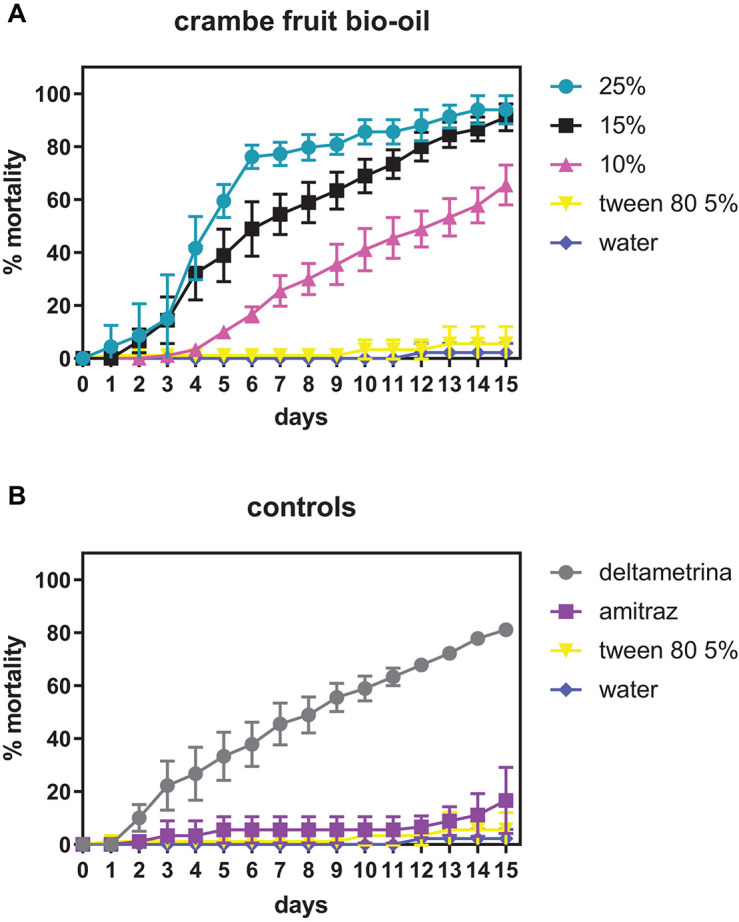
Mortality of *Rhipicephalus microplus* females exposed to different concentrations of crambe fruit bio-oil. **(A)** Crambe fruit bio-oil in different concentrations; **(B)** positive and negative controls.

Figure 1 describes the AIT results for the crambe fruit bio-oil in different concentrations showing *R. microplus* female mortality. The acaricidal effect was elevated in all concentrations tested, causing high female mortality and inhibiting oviposition. At a concentration of 15%, crambe bio-oil exhibited mortality of 91.1% on the 15th day after treatment. When used at 10% concentration (v/v), the effect of bio-oil was less than that found by the commercial acaricide deltamethrin.

In the AIT the efficacy of treatment against engorged females was also evaluated by measuring egg production. Results showed that the IO of the crambe fruit bio-oil differed significantly from the negative controls (*p* < 0.0001) at all concentrations tested. The bio-oil at a concentration of 15% showed an IO similar to the commercial acaricide amitraz (Table 5).

**TABLE 5:** Percentage of mortality, reproductive index (RI), and inhibition of oviposition (IO) of engorged females of *Rhipicephalus microplus* exposed to crambe fruit bio-oil in different concentrations.

**Treatment**	**FM (%) ± SD**	**RI ± SD**	**IO (%)**	**VE**
water	2.8 ± 4.4	0.49 ± 0.06	—	Eggs hatched
tween 80 5%	5.5 ± 6.5	0.44 ± 0.04	9.2	Eggs hatched
amitraz	16.7 ± 12.5	0.02 ± 0.04	94.5^a,b^	There was no hatching
deltamethrin	81.3 ± 5.6^a,b^	0.06 ± 0.06	81.3^a,b^	Eggs hatched
**crambe bio-oil**				
10%	65.5 ± 18.6^a,b^	0.06 ± 0.04	78.8^a,b^	There was no hatching
15%	91.1 ± 12.4^a,b^	0.02 ± 0.02	93.3^a,b^	There was no hatching
25%	94.0 ± 6.0^a,b^	0.01 ± 0.01	96.3^a,b^	There was no hatching

*FM (%): percentage of female mortality after 15 days; RI: reproductive index; IO (%): percentage of inhibition oviposition; VE: viability of eggs.*

*amitraz: 250 μg/ml, deltamethrin: 25 μg/ml.*

*^*a*^ Significant difference to the negative control (water).*

*^*b*^ Significant difference from the negative control (Tween 80 5%) (one-way ANOVA *p* = < 0.0001).*

### Effects on the Ovary of Ticks

**FIGURE 2:**
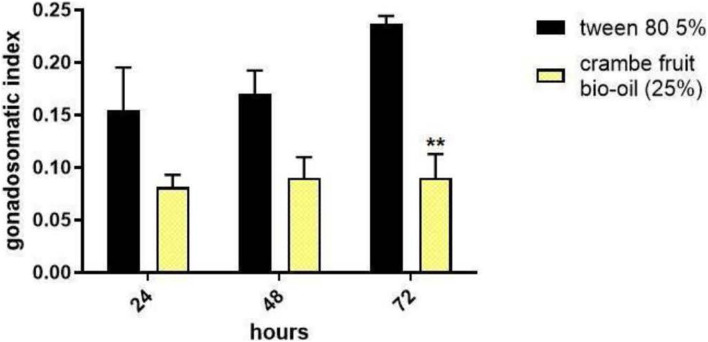
Gonadosomatic index of *Rhipicephalus microplus* tick females exposed 24, 48, and 72 h to crambe fruit pyrolysis bio-oil (25% concentration). Tween 80 (5%) was used as a negative control. The experimental number was 45 females/treatment. The results are expressed as mean ± SD, **p < 0.05 (two-way ANOVA).

To analyze the interference of crambe fruit bio-oil in ovarian development, engorged females were treated with bio-oil at a concentration of 25% and dissected 24, 48, and 72 h after treatment. Females treated with bio-oil showed a reduction in GSI in all the periods evaluated, however, only the reduction in 72 h of exposure was significant (Figure 2).

**FIGURE 3:**
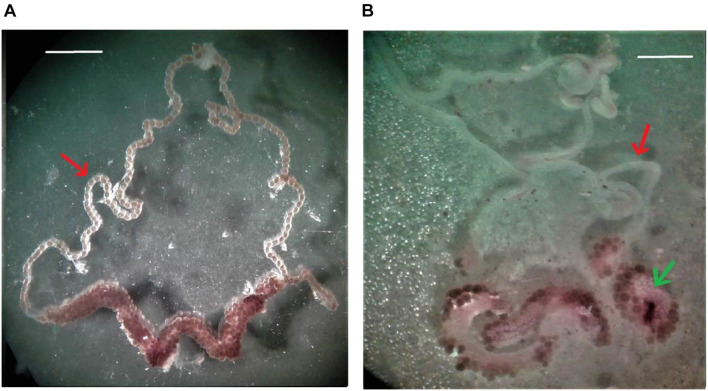
Ovaries of engorged females treated with 5% tween 80 (negative control) **(A)** and females treated with the crambe fruit pyrolysis bio-oil **(B)** obtained after 72 h of treatment. Red arrow: oviduct; green arrow: poorly developed oocytes (increase: 20×). Bar = 1 cm.

During dissection, it was observed that the ovaries of females in groups treated with crambe fruit bio-oil had small and whitish oocytes after 72 h, therefore, little developed (Figure 3). In addition, there are no mature oocytes in the oviduct, whereas in the ovary of the control group, its presence can be observed.

## Discussion

Liquid pyrolysis products have been the target of studies evaluating biocidal effects in different organisms. Bio-oils have a high yield and a rich chemical composition, which varies according to the biomass used and the conditions of the thermochemical process. Slow pyrolysis of crambe fruit gave rise to a high bio-oil yield. The general distribution of the yield of crambe fruit pyrolysis products was like that described in the literature (Silva et al., 2019).

Results of this study show that slow pyrolysis of crambe fruit produced a bio-oil with toxic effect in *R. microplus*. This is the first report demonstrating the effect of pyrolysis bio-oil as an acaricide. A previous study carried out by Lindqvist et al. (2009) evaluated the acaricide effect of a pyrolysis bio-oil (birch tar oil) against *Phytonemus pallidus* (Acari: Tarsonemidae) and *Tetranychus urticae* (Acari: Tetranychidae) mites. However, the bio-oil was not toxic to mites. Most studies evaluating the effect of pyrolysis products on arthropods have been carried out with insects and this is an area of research that has not yet been fully explored (Mattos et al., 2019).

Crambe fruit bio-oil caused high mortality of *R. microplus* larvae in 24 h, with an LC90 of 14.4%. Similarly, some bio-oils also showed the larvicidal effect on larvae of Colorado potato beetle *Leptinotarsa decemlineata* (Coleoptera: Chrysomelidae). Pyrolysis bio-oil from dried coffee grounds and tobacco-caused 100% mortality of CPB larvae in 48 h at a concentration of 50 and 100%, respectively (Booker et al., 2010; Bedmutha et al., 2011), concentrations higher than that found with the crambe bio-oil in *R. microplus*. These studies, different from ours, used fast pyrolysis at higher temperatures (400–600°C). The fast pyrolysis process at temperatures above 500°C, although generate a high yield of bio-oil (Bridgwater and Peacocke, 2000; Bridgwater, 2012), typically form polycyclic aromatic hydrocarbons (Simko, 2005; Mohan et al., 2006), toxic substances to humans and environment (EPA, 2008; Wu et al., 2011).

Crambe fruit bio-oil significantly interfered in tick reproduction, inhibiting egg development and egg-laying. At a lower concentration (10%), mortality was gradual, reaching 65.5% mortality only on the 15th day after treatment. However, on the fourth day, which would be the peak of egg-laying (Hitchcock, 1955), only 3% of the females had died, which does not explain the high inhibition of egg-laying observed. These results show that crambe fruit bio-oil, in addition to being toxic to females tick causing their death, also prevents them from laying eggs, contributing to stopping the tick cycle.

Thus, it was investigated if there was a metabolic interference, impairing the development of eggs or whether the organs associated with the egg-laying process (Genet’s organ, for example) may have been affected. Treated *R. microplus* females were dissected to analyze the ovaries. Crambe fruit bio-oil significantly reduced the GSI by more than half after 72 h. Barbosa et al. (2016) also observed a strong decrease in GSI and the number of oocytes when *R. microplus* engorged females were treated with 3β-O-tigloylmelianol, a protolimonoid isolated from *Guarea kunthiana* (Meliaceae).

According to Balashov (1983), oogenesis can be divided into five stages. Phases III and IV of oogenesis consist of the period of formation of yolk granules by oocytes, and at the end of this period, the oocyte is ready for ovulation. During ovulation (stage V), the oocyte passes through the ovary lumen, is moved by peristaltic movements to the oviduct and, finally, to the vagina (Balashov, 1983; Denardi et al., 2004). The oocytes of *R. microplus* females treated with crambe fruit bio-oil were poorly developed. As the females were incubated after feeding and unformed eggs were seen in the ovary, there may be a change in stage III or IV of oogenesis in females treated with crambe fruit bio-oil. In addition, unlike the control group, there were no oocytes in the oviduct, indicating that the oocytes did not reach the last stage of development (V). Our group also evaluated ovaries of tick females treated with pyrolysis bio-oils from other biomasses, and there was no significant reduction in GSI (data not shown).

Bio-oils have a large number of organic compounds, and this chemical composition is dependent on raw material and the process conditions used. The acaricidal activity found may be associated with a synergistic effect between the various substances present in crambe fruit bio-oil. Phenolic compounds, fatty acids, and acetic acid have been found in liquid pyrolysis products from plant biomass with important insecticidal properties (Yatagai et al., 2002; Bedmutha et al., 2011; Kartal et al., 2011; Suqi et al., 2014; Cáceres et al., 2015). However, there are still few studies evaluating the effect of pyrolysis products on arthropods, and not all have evaluated the chemical composition (Mattos et al., 2019). Crambe fruit bio-oil exhibited many hydrocarbons in its hexane fraction. Hydrocarbons have already been found as major compounds in pyrolysis bio-oils of coffee grounds and macadamia nutshells, and these bio-oils were active against CPB and the termite *Coptotermes formosanus*, respectively (Yatagai et al., 2002; Bedmutha et al., 2011).

These interesting data show us that crambe fruit pyrolysis bio-oil is a promising acaricide against the cattle tick *R. microplus*. However, additional research must be carried out to investigate issues related to environmental safety, to develop a safe product for the population.

## Conclusion

Slow pyrolysis of crambe fruits produces a bio-oil in high yield, containing several organic compounds being a good source for developing new biotechnological products. This is the first report of the use of pyrolysis bio-oil as an acaricide. The results of this study demonstrate the acaricidal effect of crambe fruit bio-oil on larvae and engorged females of *R. microplus*. In addition, bio-oil interferes with tick reproduction, inhibiting egg-laying. Crambe fruit bio-oil is, therefore, a potential alternative of sustainable product for control *R. microplus.*

## Data Availability Statement

The raw data supporting the conclusions of this article will be made available by the authors, without undue reservation.

## Ethics Statement

The animal study was reviewed and approved by the Ethics Committee on Animal Experimentation of UFRGS and FEPAGRO, Brazil.

## Author Contributions

GA, MC, and EF contributed to the conception and design of the study. CM and JA organized the database. CM performed the statistical analysis and wrote the first draft of the manuscript. CM, JA, and NT performed tick experiments. BS performed pyrolysis conversion. BS, CM, MC, and GA performed the chemical analysis. CM and EF wrote the final manuscript. All authors contributed to manuscript revision, read, and approved the submitted version.

## Conflict of Interest

The authors declare that the research was conducted in the absence of any commercial or financial relationships that could be construed as a potential conflict of interest.

## Publisher’s Note

All claims expressed in this article are solely those of the authors and do not necessarily represent those of their affiliated organizations, or those of the publisher, the editors and the reviewers. Any product that may be evaluated in this article, or claim that may be made by its manufacturer, is not guaranteed or endorsed by the publisher.
